# A novel BR-SMAD is required for larval development in barber's pole worm *Haemonchus contortus*

**DOI:** 10.15698/mic2021.02.742

**Published:** 2020-12-23

**Authors:** Fangfang Li, Peixi Qin, Lisha Ye, Nishith Gupta, Min Hu

**Affiliations:** 1 State Key Laboratory of Agricultural Microbiology, College of Veterinary Medicine, Huazhong Agricultural University, Wuhan, People's Republic of China.; 2 Department of Molecular Parasitology, Faculty of Life Sciences, Humboldt University, Berlin, Germany.; 3 Department of Biological Sciences, Birla Institute of Technology and Science Pilani (BITS-P), Hyderabad, India.

**Keywords:** Haemonchus contortus, R-SMADs, BMP subfamily, RNAi, larval development

## Abstract

SMAD proteins mediate TGF-β signaling and thereby regulate the metazoan development; however, they are poorly defined in *Haemonchus contortus*–a common blood-sucking parasitic nematode of small ruminants. Here, we characterized an R-SMAD family protein in *H. contortus* termed *Hc*SMA2, which is closely related to *Caenorhabditis elegans* SMA2 (*Ce*SMA2) involved in the bone morphogenetic protein (BMP) signaling. *Hcsma2* is transcribed in all developmental stages of *H. contortus* but highly induced in the adult male worms. The RNA interference with *Hcsma2* retarded the transition of infective L3 into L4 larvae. Besides, the bimolecular fluorescence complementation revealed the interaction of *Hc*SMA2 with a TGF-β-activated-R-SMAD (*Hc*DAF8). Together these results show a BMP-like receptor-regulated SMAD in *H. contortus* that is required for larval differentiation and underscore an adaptive functional repurposing of BMP-signaling in parasitic worms.

## INTRODUCTION

The known members of the TGF-β (transforming growth factor-β) superfamily can be classified into two main categories including the TGF-β/activin/nodal subfamily and the bone morphogenetic protein (BMP)/growth and differentiation factor (GDF)/Müllerian inhibiting substance (MIS) subfamily [1]. The classical SMAD-dependent TGF-β signaling involves the binding of an extracellular TGF-β homolog to a heterodimeric receptor complex, resulting in the activation and eventual nuclear translocation of specific cytoplasmic mediators (SMADs), which in turn regulate transcriptional activity by interacting with various transcription factors, co-activators and co-repressors [2]. In mammalian cells, such receptor-regulated SMADs (R-SMADs) are either activated by BMP type I receptors (*aka* BR-SMADs; SMAD1, SMAD5, SMAD8), or by activin and TGF-β type I receptors (*aka* AR-SMADs; SMAD2, SMAD3) [3]. TGF-β signaling also regulates the development of other metazoans including parasitic and free-living nematodes as described below.

*Caenorhabditis elegans* expresses five ligands of the TGF-β superfamily, of which DAF7 and DBL1 (Dpp and BMP-like-1), which belong to a canonical receptor-SMAD pathway, have been well studied. While DAF7 signaling is an important determinant of both dauer entry and exit decision [4], the DBL1 (Dpp and BMP-like-1) pathway regulates body size, male tail morphogenesis, cell lineage decision and innate immunity [5]. The DBL1 signal is relayed by a heterotetrameric receptor complex that is composed of two SMA6 type I and two DAF4 type II receptor subunits. The type II receptors first phosphorylate and activate the type I receptors, which in turn phosphorylate the downstream R-SMADs (SMA2 and SMA3). Phosphorylated R-SMADs interact with Co-SMAD (common-mediator SMAD; SMA4) and translocate to the nucleus for transcriptional regulation. Transcription factors that act with SMADs to exert DBL1-mediated responses include SMA9/Schnurri, LIN31/forkhead, and MAB31 [6]. Interestingly, the DBL1 and DAF7 pathways have some functional overlap in maintaining a non-dauer animal [7]. Both ligands (DBL1 and DAF7) interact with a sole type II receptor DAF4, and the specificity is determined by different type I receptors.

In *Haemonchus contortus,* previous work demonstrating the important roles of DAF7 signaling during larval development of this economically important nematode parasite has focused on TGF-β ligand [8], TGF-β type I receptor [9], TGF-β type II receptor [10]), TGF-β-regulated R-SMAD [11] and Co-SMAD [12]. Little is known about BMP (DBL1) signaling in this parasite, however. Here, we identified and characterized a new member of the *H. contortus* R-SMAD family (*Hc*SMA2), which interacts with *Hc*DAF8 (an R-SMAD) and contributes to the larval development.

## RESULTS AND DISCUSSION

### *Hc*SMA*2* is a member of the BMP-activated R-SMAD subfamily

**Figure 1:**
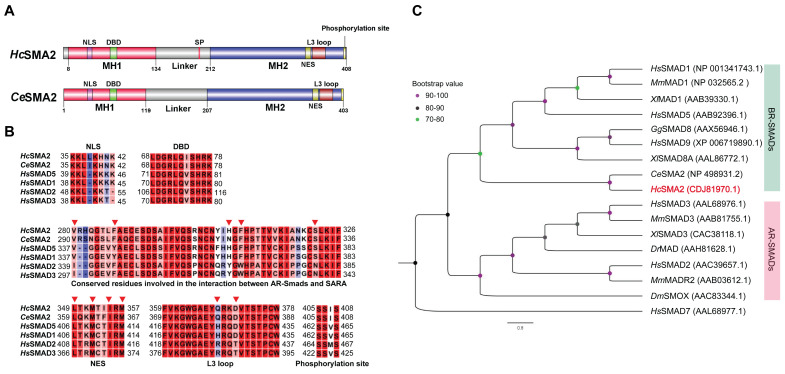
FIGURE 1: *HcSMA2 displays conserved features of the BR-SMAD subfamily*. **(A)** Primary structures of *Hc*SMA2 and *Ce*SMA2 with signature domains and motifs. **(B)** Sequence alignment of *Hc*SMA2 with representative members of R-SMADs. The functional motifs include nuclear localization signal (NLS), DNA-binding β-hairpin domain (DBD), nuclear export signal (NES), L3 loop with R-SMAD subtype-specific amino acid residues, and phosphorylation motif. Residues involved in the interaction between R-SMAD and SARA as well as NES are highlighted (▾). The NCBI GenBank accession: *Hc*SMA2, CDJ81970.1; *Ce*SMA2, NP_498931.2; *Hs*SMAD5, AAB92396.1; *Hs*SMAD1, NP_001341743.1; *Hs*SMAD2, AAC39657.1; *Hs*SMAD3, AAL68976.1. **(C)** Phylogenetic analysis of *Hc*SMA2 and selected homologs from *Danio rerio, Gallus gallus, Homo sapiens, Mus musculus* and *Xenopus laevis, Drosophila melanogaster* and *C. elegans*. The eventual cladogram was rooted using the inhibitory SMAD from *H. sapiens* SMAD7. Nodal support values for each clade are color-coded, and the accession numbers of individual proteins are listed in brackets next to each protein.

Our BLAST search revealed the presence of *Hc*SMA2 protein in *H. contortus*, which shares 72% identity to *Ce*SMA2. *Hc*SMA2 (408 aa) contains conserved MH1 (mad homology domain; 8-134 aa) and MH2 (212-408 aa) domains along with signature motifs and residues of an R-SMAD (**Fig. 1A**). A nuclear localization signal and a DNA-binding β-hairpin domain were identified in the MH1 domain [13]. The proline-rich linker region contains an SP motif that may be phosphorylated by Serine/Threonine kinases [14]. Likewise, the MH2 domain harbors a conserved L3 loop, albeit Q^368^ and D^371^ that determine the interaction between R-SMADs and receptors diverge from that of BR-SMADs [15, 16]. The VYHFS and IFYWN motifs regulate the interaction of R-SMADs and SARA (SMAD-anchoring for receptor activation) [17] in BR-SMADs and AR-SMADs, respectively. Similar to BR-SMADs, the VYHFS is broadly preserved in *Hc*SMA2 (V^280^, H^307^, F^309^, S^322^; **Fig. 1B**). Specific residues (L^349^, M^352^, I^355^, M^357^) of the nuclear export signal (NES) could also be found in *Hc*SMA2 [17]. Moreover, a typical SSxS motif for phosphorylation by type I receptor is present at the extreme carboxyl terminus (**Fig. 1B**), which is a hallmark of the R-SMAD subfamily [18]. Not least, the phylogenetic analysis revealed a clading of *Hc*SMA2 and *Ce*SMA2 along with other members of the BR-SMAD group (**Fig. 1C**).

### *Hc*SMA*2* transcript is upregulated in the adult male worms

**Figure 2:**
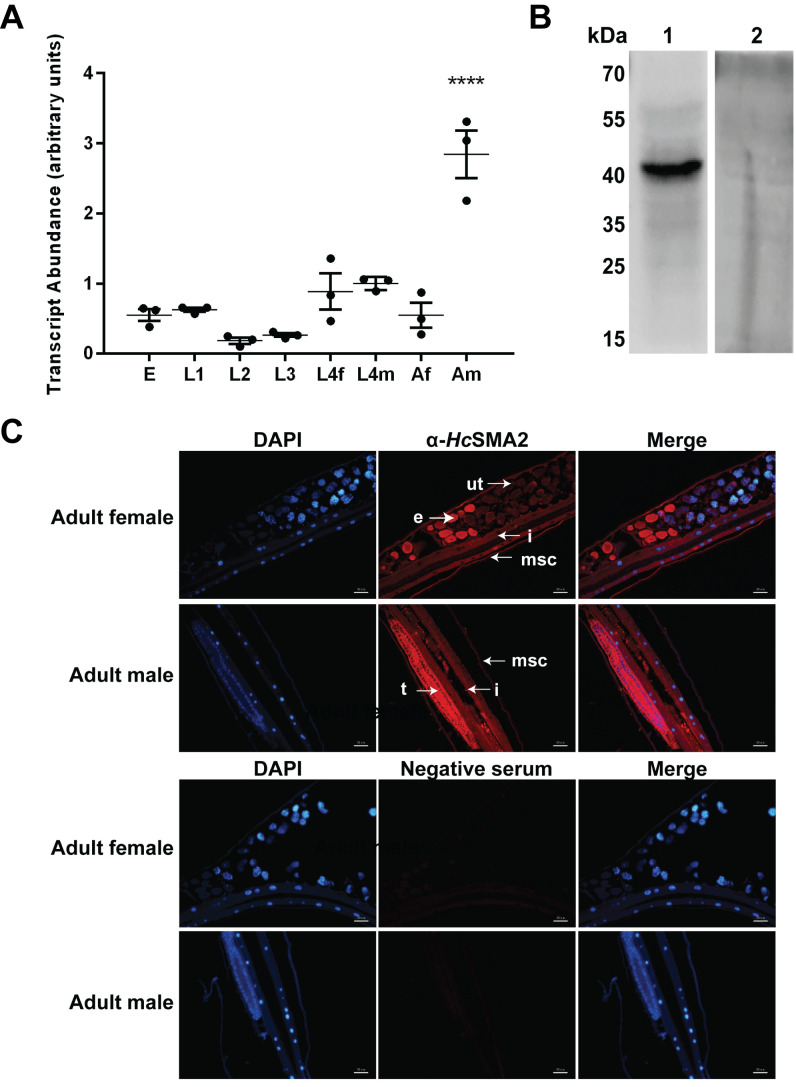
FIGURE 2: *Spatiotemporal expression of HcSMA2 in H. contortus*. **(A)** Transcriptional profile of *Hcsma2* in eggs (E), the first-stage larvae (L1), the second-stage larvae (L2), the third-stage larvae (L3), the fourth-stage female (L4f), the fourth-stage male (L4m), adult female (Af), and adult male (Am) worms. Data show mean ± SEM from 3 assays (****, *p* ≤0.0001). **(B)** Western blot of the total worm protein probed using the rabbit anti-*Hc*SMA2 serum (lane 1) and the pre-immune rabbit serum (lane 2). **(C)** Tissue distribution of *Hc*SMA2 in adult worms. Immunofluorescence was detected by α-*Hc*SMA2 and pre-immune sera. Organs annotated in the image include platymyrian muscle cells under cuticle (msc), eggs (e), uterus (ut), intestine (i), and testis (t). *Scale bar*, 50 μm.

We examined the transcriptional profile of *Hc*SMA2 during different developmental stages of *H. contortus* by RT-PCR and its tissue distribution in the adult worms by immunofluorescence assay (**Fig. 2**). *Hcsma2* was transcribed in all stages with the highest transcript abundance in adult males (**Fig. 2A**). Next, we generated a polyclonal antibody against a synthetic partial polypeptide of *Hc*SMA2, which recognized a band of the expected size (46-kDa) in the total parasite lysate (**Fig. 2B**). Immunofluorescence assay disclosed that *Hc*SMA2 was expressed in the platymyrian muscle cells under the cuticle and in the intestinal cytoplasm of both female and male adults, as well as in eggs within the female worms, and in the testis of the male worms (**Fig. 2C**). The distribution of *Hc*SMA2 contrasts with *Ce*SMA2 in *C. elegans*, which is mainly expressed in the pharynx, reproductive system, and head cells of adult worms [19, 20]. However, the overlapping expression of *Hc*SMA2 and *Hc*TGFBR2 [10] in the reproductive system and high transcript level of both in the adult male worms indicate that *Hc*SMA2 may interact with *Hc*TGFBR2 by conveying the BMP signals during spermatogenesis and embryonic development [21]. The expression of *Hc*SMA2 in the platymyrian muscle cells and intestine also indicates its functions in muscle movement, invasion, and immunity [22].

### *Hc*SMA2, interacting with *Hc*DAF8, is required for the worm development

**Figure 3:**
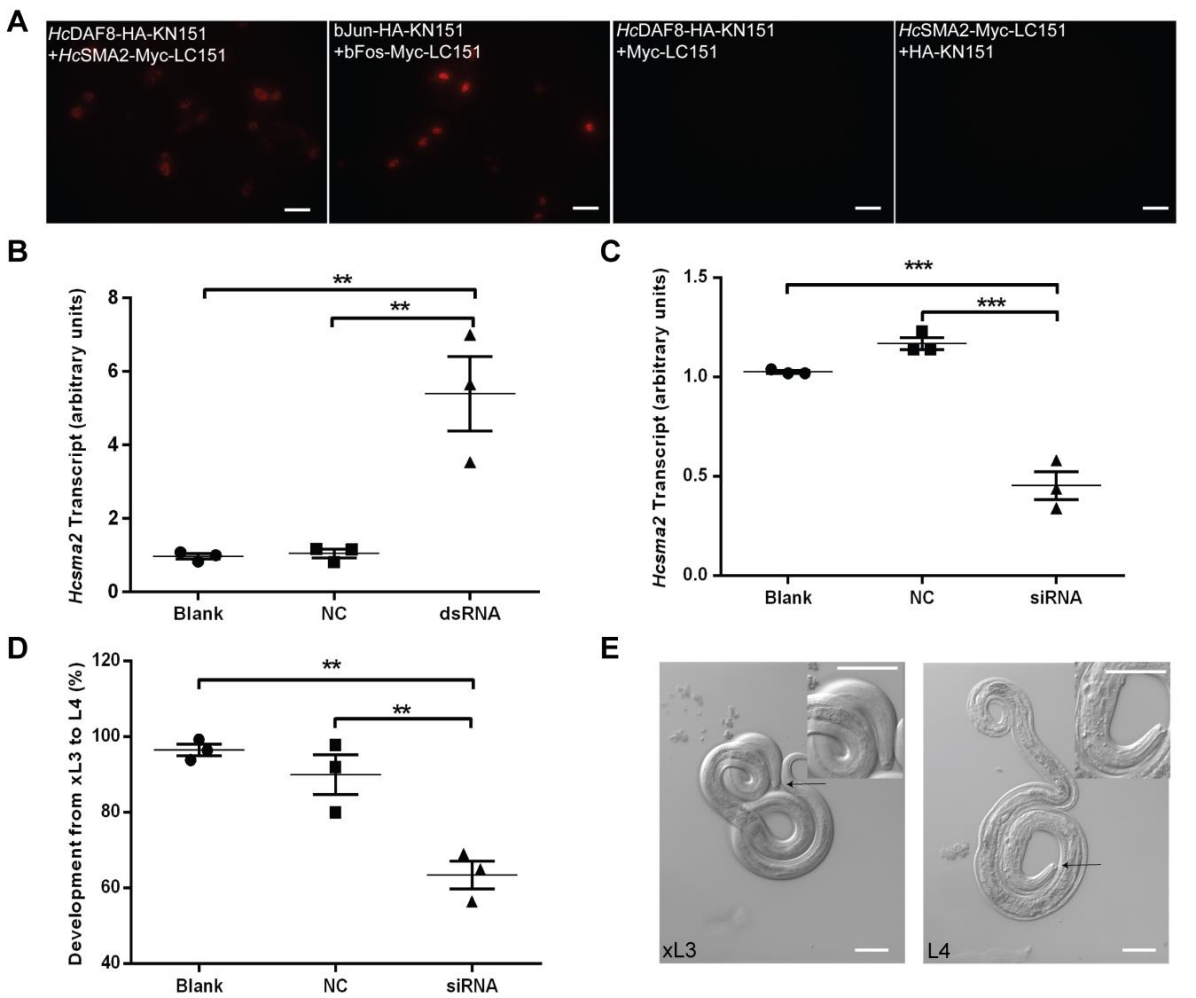
FIGURE 3: *HcSMA2 interacts with HcDAF8 and is involved in differentiation of xL3 to L4 larvae.* **(A)** Bimolecular fluorescence complementation revealing close proximity of *Hc*SMA2 and *Hc*DAF8 in BHK21 cells. *Scale bar*, 20 μm. **(B)** Transcript abundance of *Hcsma2* in worms transfected with *Hcsma2*-specific dsRNA, negative control (NC, *Btcry1Ac*-specific dsRNA), or blank (EBSS). **(C)** Transcript levels of *Hcsma2* in worms treated with *Hcsma2*-specific siRNA, negative control (NC, negative siRNA), or blank (EBSS). **(D)** The percentage of L4 larvae developed from xL3 after siRNA-treatment, as shown in *panel C*. Graphs in *panel B-D* show means ± SEM from 3 assays (**, *p* ≤0.01; ***, *p* ≤0.001). **(E)** The morphology of the mouth capsule of xL3 and L4 stage larvae. *Scale bar*, 50 μm.

Our further work investigated whether *Hc*SMA2 interacts with *Hc*DAF8 [11] via a bimolecular fluorescence complementation assay (**Fig. 3A**). As a positive control, we transfected BHK21 cells with plasmid constructs encoding for bJun-HA-KN151 and bFos-Myc-LC151 fusion proteins, which exhibited a red signal in the nucleolus. Equally, cells co-transfected with *Hc*SMA2-Myc-LC151 and *Hc*DAF8-HA-KN151 constructs displayed red fluorescence in the cytoplasm. No fluorescence in cells harboring *Hc*DAF8-HA-KN151 and Myc-LC151 proteins, or *Hc*SMA2-Myc-LC151 and HA-KN151 protein pair was observed. These results indicated that *Hc*SMA2 interacts with *Hc*DAF8, and possibly regulate DAF7 signaling during the parasite development.

Last but not least, we examined the physiological importance of *Hc*SMA2 in *H. contortus* by double strand RNA (dsRNA) or small interfering RNA (siRNA)-mediated RNA interference (**Fig. 3B-E**). Surprisingly, soaking of exsheathed L3 (xL3) larvae in dsRNA targeting the *Hcsma2* caused a notable up-regulation of its transcript and did not exert a detectable phenotype (**Fig. 3B**). In contrary, *Hcsma2* was silenced efficiently when the xL3 larvae were soaked in gene-specific siRNA (**Fig. 3C**), which resulted in a significant decline in morphogenesis to L4 larvae when compared to the control siRNA, and the blank control group (**Fig. 3D**). The morphological difference in the buccal capsule of xL3 and L4 larvae was also quite apparent, where the latter had its buccal capsule as an inverted cone and a pair of symmetric structures (mouthparts; **Fig. 3E**). These results suggest a role of *Hc*SMA2 in differentiation of infective xL3 to L4 larvae, which is consistent with the function of the known members of the TGF-β and Insulin signaling [8–12, 23]. In *C. elegans, Ce*SMA2 regulates the body size [24], morphogenesis of the male worm's tail tip [25], as well as reproductive aging [21, 26]. In contrast, *Hc*SMA2 displays a notable functional divergence of the BMP-signaling. In conclusion, our work reveals a BMP-like receptor-regulated SMAD in *H. contortus* required for larval differentiation which has been repurposed to the parasitic lifestyle.

## MATERIALS AND METHODS

### Maintenance of *H. contortus*

The Haecon-5 strain of *H. contortus* was maintained by serial passages in experimental goats (12-14 weeks of age; raised helminth-free), as described previously [27]. Animals were infected orally with infective L3 stage larvae (7000 iL3s/kid). Eggs isolated from the goat feces by sucrose flotation [28] were cultured in a nutritive medium (0.1 mL of 1x Earle's balanced salt solution (Sigma-Adrich) and 0.5% (w/v) yeast extract) at 28°C [29]. The L1, L2, and iL3 stages were obtained from eggs cultured at 28°C for 1, 4 and 7 d, respectively [30]. The L4 stages and adults of both sexes were collected from the abomasum of the infected goats euthanized 8 and 30 d post-inoculation with iL3s [27]. Males and females of the L4 and adult stages were separated as reported earlier [31]. All experimental animals used in this project were treated in accordance with the protocol approved by the Ethics Committee of Huazhong Agriculture University, Wuhan, People's Republic of China (HZAUGO-2016-007).

### Isolation of *Hs*SMA2 in *H. contortus*

Homologs of *Ce*SMA2 (WormBase, WS271, code ZK370.2) were identified in *H. contortus* (PRJEB506, PRJNA205202) (https://wormbase.org/tools/blast_blat) [32]. Using genomic and transcriptomic datasets for *H. contortus* [33], the coding sequence of *Hc*SMA2 was isolated (GenBank: HF957107.1). In brief, the adult worms were homogenized, lysed with the TRIpure reagent (Aidlab, Beijing), and total RNA was extracted according to the manufacturer. The quality and yield of RNA were verified by electrophoresis and spectrophotometry (NanoDrop Technologies, USA). First-strand cDNA synthesis was performed by the Maxima H Minus First-Strand cDNA Synthesis Kit (Thermo Scientific, USA). The open reading frame of *Hc*SMA2 was amplified from the first-strand cDNA using *Hc*SMA2-F and *Hc*SMA2-R primers (Table S1).

### Bioinformatic analysis

The protein sequence of *Hc*SMA2 (GenBank: CDJ81970.1) was analyzed for the conserved residues and motifs using Pfam (www.sanger.ac.uk/Software/Pfam), PROSITE (http://prosite.expasy.org) databases and CLC genomics workbench. The nuclear localization signal (NLS) was predicted by cNLS Mapper (http://nls-mapper.iab.keio.ac.jp/cgi-bin/NLS_Mapper_form.cgi). Phylogenetic clading with representative members of the SMAD family was performed using the Maximum Likelihood (ML) method with 1,000 bootstrap replications.

### Preparation of *Hc*SMA2 antibody and immunoblot analysis

A synthetic peptide antigen (CDLQSHHELKAID) corresponding to 94-105 aa of *Hc*SMA2-MH1 domain was used to immunize rabbits (Willget Biotech Co. Ltd., Shanghai, China). The *Hc*SMA2 antiserum was tested by Western blot analysis of total protein extracted from the adult worms using a commercial kit (BestBio, China). Proteins were resolved by 12% sodium dodecyl sulfate-polyacrylamide gel electrophoresis (SDS-PAGE) and transferred onto an Immobilon^®^-PSQ membrane (Merck Millipore Ltd). The immunoblot membranes were blocked with blocking buffer (1% (w/v) BSA (BioFROXX, Guangzhou, China) in PBS, 20% Tween-20; PBST) for 6 h at 4°C, washed 3 times with PBST and incubated with the *Hc*SMA2 antiserum (1:1000 in PBST) overnight at 4°C. Samples were washed 6x in PBST and subsequently incubated with an HRP-conjugated goat anti-rabbit antibody (1:1000, Beyotime Biotechnology, China) for 2 h at 37°C, followed by 5x additional washes. Immunodetection was performed by chemiluminescence (WesternBright ECL kit; K-12045-D10, Aibio, China), and images were acquired by ChemiDoc XRS+ system (Bio-Rad, USA).

### Indirect immunofluorescent assay

Fresh adult worms were washed with PBS (pH 7.4) and fixed overnight using 4% paraformaldehyde in PBS at 4°C [34]. Samples were washed, dehydrated, immersed in paraffin wax, and sliced. Sections (4 μm) were incubated in 3% hydrogen peroxide for 10 min at room temperature (24°C) to quench the endogenous peroxidase activity. They were pre-blocked with BSA for 20 min at 37°C, and then probed overnight with the *Hc*SMA2 antiserum (1:100 in PBST) at 4 °C. The “negative control” sections were probed with the pre-immune rabbit serum (same dilution). Following three washes (5 min each), sections were incubated with Alexa Fluor^®^ 594 goat anti-rabbit antibody (1:3000, ThermoFisher Scientific, R37117) for 50 min at 37°C. Unbound secondary antibody was washed off 3x by PBST, and samples were incubated with 4′,6-diamidino-2-phenylindole (DAPI) for 5 min at 24°C to visualize nuclei. Subsequently, samples were washed and mounted for fluorescent imaging (Olympus, BX51, Japan).

### Double-strand RNA (dsRNA)-mediated RNA interference

The gene-specific dsRNA was synthesized as described elsewhere [9]. A fragment encoding partial MH2 domain (630-1220 bp) was amplified using *Hc*sma2i-F1/R1 (tagged with a T7 promoter site in the forward direction, and *Hind*III restriction enzyme site in the reverse direction) and *Hc*sma2i-F2/R2 (tagged with a *Hind*III restriction site (forward) and a T7 promoter site (reverse)) primer pairs (Table S1). Plasmids were linearized by *Hind*III and examined by agarose electrophoresis to test the enzymatic cleavage. Linearized DNA was gel-purified using Axygen^®^ AxyPrep PCR Clean-Up kit (Axygen, Hangzhou, China), and precipitated in 2 volumes of 100% ethanol and 0.1 volume of 3 M sodium acetate (-20°C for 7 h). The DNA pellet was collected by centrifugation, washed with 75% ethanol, air-dried, resuspended in sterile water, and measured by spectrophotometer (NanoDrop Technologies, USA). The eventual DNA preparation was used as the template for *in vitro* transcription reactions using the MEGAscript^®^ T7 transcription kit (Ambion, USA). The complementary RNA strands were annealed by mixing equimolar amounts and heated to 90°C for 5 min, followed by a further 2 h incubation at 37°C. The quality and concentration of the dsRNA were measured by electrophoretic analysis and spectrophotometry. Two sequences of the *cry1Ac* gene from *Bacillus thuringiensis* (*Btcry1Ac*; GenBank: GU322939.1) designed to produce the complementary single RNA strands (negative control) were amplified using *Bt*cry1Ac-F1/R1 and *Bt*cry1Ac-F2/R2 primers (Table S1). This fragment showed no significant homology with any nematode sequence in public databases [9]. The synthesis of dsRNA of *Btcry1Ac* was completed as described above. The dsRNA was stored at -80°C (80 µg/tube).

Specific dsRNAs encapsulated in a liposome preparation were added to worms. In brief, iL3s (10,000 larvae/group) were exsheathed with 0.15% sodium hypochlorite/PBS at 38°C until most of the shed sheaths were visible under the microscope (*ca.* 30 min), followed by 6x washes with PBS and 4x washes in Earle's balanced salt solution (EBSS, Sigma; pH adjusted to 5.2). The exsheathed L3 (xL3) larvae were soaked in 80 μL of EBSS. The liposome formulation of dsRNA was prepared by gentle mixing in a final volume of 20 μL (1 μL of lipofectamine 2000 (Invitrogen), 1 μL of RNasin (Promega), 80 µg of dsRNA and the residual volume of EBSS), allowed to stand for 10 min at room temperature and then added to the larvae (final concentration of dsRNA, 1 µg/µL). The larvae were incubated for 24 h at 37°C under 20% (v/v) CO_2_. Approximately, 200 larvae of each group were transferred to fresh EBSS culture medium for further 6 d to assess their developmental level and the remaining larvae were washed separately in a 10 mL glass tube with PBS for 4 times. The sedimented worms (8,000*g* for 4 min) were frozen in Trizol (Aidlab, Beijing, China) at -80°C for nucleic acid preparation and transcript analysis. On the 7^th^ day, the worms that developed to the L4 stage were counted according to the phenotype of the buccal capsule [35, 36].

### Small interfering RNA (siRNA)-mediated RNA interference

Three custom siRNA in different segments of *Hcsma2* were synthesized based on the gene coding sequence (GenePharma, Shanghai, China) (Table S2). The “negative control” siRNA lacking any homology with *H. contortus* sequence in public databases was also synthesized (Table S2). All siRNA preparations were stored in DEPC (50 μM)-treated water at −20°C. The clean exsheathed L3 worms were soaked in 80 μL of EBSS. Lipofectamine 2000 (5 μL) was mixed gently with 8.8 of μL EBSS and allowed to incubate for 5 min at room temperature followed by additional incubation with 6 μL of siRNA (siRNA-136 + siRNA-625 + siRNA-1130, 1 μM each) and 0.2 μL RNasin (8 U) for 15 min. The liposome formulation of siRNA (20 μL) was added into the exsheathed L3 cultures with a final volume of 100 μL in each well. The negative and blank controls were set up with 6 μL of “negative control” siRNA (3 μM) or 6 μL EBSS, respectively. Larvae were incubated at 37°C, 20% CO_2_ for 3 d. Approximately, 200 larvae of each group were transferred to fresh EBSS culture medium with 5% fetal bovine serum (Gibco, China) for another 4 d to assess their development. The remaining larvae were subjected to RNA extraction. The L4 larvae were counted on the 7^th^ day.

### Real-time PCR

RT-PCR was employed to examine the relative transcriptional abundance of *Hcsma2* in different developmental stages (eggs, L1s, L2s, iL3s, L4f, L4m, Af and Am) of *H. contortus* (Haecon-5 strain) using the primers rt*Hc*sma2-F and rt*Hc*sma2-R (Table S1). Briefly, RNA was isolated from collected samples using TRIpure Trizol (Aidlab, Beijing, China). 1 μg RNA of the each stage was used to make the first-strand cDNA using HiScript III-RT SuperMix (Vazyme, Nanjing, China, R323-01). RT-PCR (10 μL) was set up using the ChamQ Universal SYBR qPCR Master Mix (Vazyme, Nanjing, China, Q711-02) in a thermal cycler (QuantStudio 3, Thermo Fisher Scientific, USA) under the following conditions: 50°C/2 min and 95°C/30 sec for the first cycle, then 95°C/15 sec, 60°C/15 sec and 72°C/20 sec for 40 cycles. The dissociation curve was generated under the following conditions: 95°C/15 sec, 60°C/1 min, and 95°C/1 sec. Each sample was tested in triplicate, employing β-tubulin (Accession ID, M76493) as a reference gene and corresponding primers (Tubulin-F/R, Table S1) [37]. The mean threshold cycle (*Ct*) values were subjected to estimate the relative quantities with respect to L4f (L4f = 1) using the 2-^ΔΔCt^ method [38]. Besides, the transcripts of *Hcsma2* in the RNAi-treated and control worms were quantified using RT-PCR. Here, the *Hc18S* rRNA gene (primers in Table S1) was used as a control as reported [39]. Fold change expression of *Hcsma2* after RNAi calculated by 2-^ΔΔCt^ method. ΔΔ*Ct* = [(*Ct*_*RNAi,Hcsma2*_)-(*Ct*_*RNAi,Hc18S*_)]-[(*Ct*_*Blank,Hcsma2*_)-(*Ct*_*Blank,Hc18S*_)].

### Bimolecular fluorescence complementation assay (BiFC)

The bimolecular fluorescence complementation assay was performed using two plasmids as described in a previous study [40]. The full-length cDNA of *Hc*DAF8 [11] and *Hc*SMA2 were amplified with the primers *Hc*daf8-HA-F/R and *Hc*sma2-Myc-F/R, respectively (Table S1). The plasmids pbJun-HA-KN151 and pbFos-Myc-LC151 provided by Yifan Wang [40] were digested by *Nhe*I/*Xho*I and *Nhe*I/*Pvu*I, respectively. PCRs or digested products were purified (D2500-02, Omega, Shanghai, China) and sequenced. The amplicon of *Hcsma2* was cloned into pbFos-Myc-LC151 between *Nhe*I and *Pvu*I (replacing bFos) to generate the p*Hc*SMA2-Myc-LC151 vector. Equally, the amplicon of *HcDAF8* was cloned into pJun-HA-KN151 between *Nhe*I and *Xho*I sites (replacing bJun) to generate the p*Hc*DAF8-HA-KN151 construct by homologous recombination kit (ClonExpress Entry One-Step Cloning Kit, Vazyme, Nanjing, China). Plasmids were extracted from *E. coli* (Plasmid Mini Kit II, D6950-02, Omega, Shanghai, China).

To transfect BiFC constructs, BHK21 cells were grown to 70~80% confluency in 6-well plates. p*Hc*DAF8-HA-KN151 and p*Hc*SMA2-Myc-LC151 were co-transfected in a ratio of 1:1 (2.5 μg each) using Lipofectamine 2000 (Invitrogen). Transfected cells were incubated for 20 h (37°C, 5% CO_2_) and subsequently imaged (Olympus BX51, Japan; *Excitation*, 543 nm; *Emission*, 580-680 nm). As a positive control, constructs expressing bJun and bFos (pbJun-HA-KN151 and pbFos-Myc-LC151) were utilized, whereas p*Hc*DAF8-HA-KN151 and pMyc-LC151 or p*Hc*SMA2-Myc-LC151 and pHA-KN151 plasmid pairs served as the negative controls.

### Statistical analysis

Statistical analysis was performed using one-way ANOVA and *p*-values are calculated by Tukey's test (*, *p* ≤0.05; **, *p* ≤0.01; ***, *p* ≤0.001; ****, *p* ≤ 0.0001).

## SUPPLEMENTAL MATERIAL

Click here for supplemental data file.

All supplemental data for this article are available online at http://www.microbialcell.com/researcharticles/2020a-li-microbial-cell/.

## Abbreviations:

AR-SMAD: – activin/TGF-β/nodal activated R-SMAD,
BMP: – bone morphogenic protein,
BR-SMAD: – BMP and differentiation factor/Müllerian inhibiting substance activated R-SMAD,
Co-SMAD: – common-mediator SMAD,
DBL1: – Dpp and BMP-like-1,
dsRNA: – double strand RNA,
MH: – mad homology domain,
NLS: – nuclear localization sequence,
R-SMAD: – receptor-regulated SMAD,
siRNA: – small interfering RNA,
TGF-β: – transforming growth factor β.

## References

[1] Miyazawa K, Shinozaki M, Hara T, Furuya T, Miyazono K (2002). Two major Smad pathways in TGF-beta superfamily signalling.. Genes Cells.

[2] Massagué J (2012). TGFβ signalling in context.. Nat Rev Mol Cell Biol.

[3] Miyazono K, Maeda S, Imamura T (2005). BMP receptor signaling: transcriptional targets, regulation of signals, and signaling cross-talk.. Cytokine Growth Factor Rev.

[4] Thomas JH, Birnby DA, Vowels JJ (1993). Evidence for parallel processing of sensory information controlling dauer formation in *Caenorhabditis elegans*.. Genetics.

[5] Patterson GI, Padgett RW (2000). TGF beta-related pathways. Roles in *Caenorhabditis elegans* development.. Trends Genet.

[6] Gumienny TL, Savage-Dunn C (2013). TGF-β signaling in *C. elegans*.. WormBook..

[7] Liu T, Zimmerman KK, Patterson GI (2004). Regulation of signaling genes by TGFβ during entry into dauer diapause in *C. elegans*.. BMC Dev Biol.

[8] He L, Liu H, Zhang BY, Li FF, Di WD, Wang CQ, Zhou CX, Liu L, Li TT, Zhang T, Fang R, Hu M (2020). A *daf-7*-related TGF-β ligand (*Hc-tgh-2*) shows important regulations on the development of *Haemonchus contortus*.. Parasit Vectors.

[9] He L, Gasser RB, Korhonen PK, Di WD, Li FF, Zhang HR, Li FC, Zhou YQ, Fang R, Zhao JL, Hu M (2018). A TGF-β type I receptor-like molecule with a key functional role in *Haemonchus contortus* development.. Int J Parasitol.

[10] He L, Gasser RB, Li TT, Di WD, Li FF, Zhang HR, Zhou CX, Fang R, Hu M (2019). A TGF-β type II receptor that associates with developmental transition in *Haemonchus contortus in vitro*.. PLoS Negl Trop Dis.

[11] Li FF, Gasser RB, Liu F, Shan JN, Di WD, He L, Zhou CX, Wang CQ, Fang R, Hu M (2020). Identification and characterization of an R-Smad homologue (*Hco*-DAF-8) from *Haemonchus contortus*.. Parasit Vectors.

[12] Di WD, Liu L, Zhang T, Li FF, He L, Wang CQ, Ahmad AA, Hassan M, Fang R, Hu M (2019). A DAF-3 co-Smad molecule functions in *Haemonchus contortus* development.. Parasit Vectors.

[13] Dennler S, Huet S, Gauthier JM (1999). A short amino-acid sequence in MH1 domain is responsible for functional differences between Smad2 and Smad3.. Oncogene.

[14] Kretzschmar M, Doody J, Massague J (1997). Opposing BMP and EGF signalling pathways converge on the TGF-beta family mediator Smad1.. Nature.

[15] Chen YG, Hata A, Lo RS, Wotton D, Shi Y, Pavletich N, Massague J (1998). Determinants of specificity in TGF-beta signal transduction.. Genes Dev.

[16] Lo RS, Chen YG, Shi Y, Pavletich NP, Massague J (1998). The L3 loop: a structural motif determining specific interactions between SMAD proteins and TGF-beta receptors.. EMBO J.

[17] Wu G, Chen YG, Ozdamar B, Gyuricza CA, Chong PA, Wrana JL, Massague J, Shi Y (2000). Structural basis of Smad2 recognition by the Smad anchor for receptor activation.. Science.

[18] Attisano L, Lee-Hoeflich ST (2001). The Smads.. Genome Biol.

[19] McKay SJ, Johnsen R, Khattra J, Asano J, Baillie DL, Chan S, Dube N, Fang L, Goszczynski B, Ha E, Halfnight E, Hollebakken R, Huang P, Hung K, Jensen V, Jones SJM, Kai H, Li D, Mah A, Marra M, McGhee J, Newbury R, Pouzyrev A, Riddle DL, Sonnhammer E, Tian H, Tu D, Tyson JR, Vatcher G, Warner A (2003). Gene expression profiling of cells, tissues, and developmental stages of the nematode *C. elegans*.. Cold Spring Harb Symp Quant Biol.

[20] Hunt-Newbury R, Viveiros R, Johnsen R, Mah A, Anastas D, Fang L, Halfnight E, Lee D, Lin J, Lorch A, McKay S, Okada HM, Pan J, Schulz AK, Tu D, Wong K, Zhao Z, Alexeyenko A, Burglin T, Sonnhammer E, Schnabel R, Jones SJ, Marra MA, Baillie DL, Moerman DG (2007). High-throughput in vivo analysis of gene expression in *Caenorhabditis elegans*.. PLoS Biol.

[21] Luo S, Kleemann GA, Ashraf JM, Shaw WM, Murphy CT (2010). TGF-β and insulin signaling regulate reproductive aging via oocyte and germline quality maintenance.. Cell.

[22] Chisholm AD, Xu S (2012). The Caenorhabditis elegans epidermis as a model skin. II: differentiation and physiological roles.. Wiley Interdiscip Rev Dev Biol.

[23] Di WD, Gasser RB, He L, Li FF, Liu XF, Zhou CX, Zhou YQ, Fang R, Zhao JL, Hu M (2020). A serine/threonine-specific protein kinase of *Haemonchus contortus* with a role in the development.. FASEB J.

[24] Watanabe N, Ishihara T, Ohshima Y (2007). Mutants carrying two sma mutations are super small in the nematode *C. elegans*.. Genes Cells.

[25] Nelson MD, Zhou E, Kiontke K, Fradin H, Maldonado G, Martin D, Shah K, Fitch DHA (2011). A bow-tie genetic architecture for morphogenesis suggested by a genome-wide RNAi screen in *Caenorhabditis elegans*.. PLoS Genet.

[26] Luo S, Shaw WM, Ashraf J, Murphy CT (2009). TGF-beta Sma/Mab signaling mutations uncouple reproductive aging from somatic aging.. PLoS Genet.

[27] Li FC, Lok JB, Gasser RB, Korhonen PK, Sandeman MR, Shi D, Zhou R, Li X, Zhou YQ, Zhao JL, Hu M (2014). *Hc-daf-2* encodes an insulin-like receptor kinase in the barber's pole worm, *Haemonchus contortus*, and restores partial dauer regulation.. Int J Parasitol.

[28] Zawadzki JL, Kotze AC, Fritz JA, Johnson NM, Hemsworth JE, Hines BM, Behm CA (2012). Silencing of essential genes by RNA interference in *Haemonchus contortus*.. Parasitology.

[29] Rossanigo CE, Gruner L (1991). Accuracy of two methods for counting eggs of sheep nematode parasites.. Vet Parasitol.

[30] Nikolaou S, Hartman D, Presidente PJ, Newton SE, Gasser RB (2002). HcSTK, a *Caenorhabditis elegans* PAR-1 homologue from the parasitic nematode, *Haemonchus contortus*.. Int J Parasitol.

[31] Veglia F (1916). The anatomy and life-history of *Haemonchus contortus* (Rud.). Vet Res.

[32] Ma G, Wang T, Korhonen PK, Stroehlein AJ, Young ND, Gasser RB (2019). Dauer signalling pathway model for *Haemonchus contortus*.. Parasit Vectors.

[33] Schwarz EM, Korhonen PK, Campbell BE, Young ND, Jex AR, Jabbar A, Hall RS, Mondal A, Howe AC, Pell J, Hofmann A, Boag PR, Zhu XQ, Gregory T, Loukas A, Williams BA, Antoshechkin I, Brown C, Sternberg PW, Gasser RB (2013). The genome and developmental transcriptome of the strongylid nematode *Haemonchus contortus*.. Genome Biol.

[34] Skinner TM, Bascal ZA, Holden-Dye L, Lunt GG, Wolstenholme AJ (1998). Immunocytochemical localization of a putative inhibitory amino acid receptor subunit in the parasitic nematodes *Haemonchus contortus* and *Ascaris suum*.. Parasitology.

[35] Mapes CJ (1969). The development of *Haemonchus contortus in vitro*. I. The effect of pH and pCO2 on the rate of development to the fourth-stage larva.. Parasitology.

[36] Sommerville RI (1966). The development of *Haemonchus contortus* to the fourth stage *in vitro*.. J Parasitol.

[37] Guo XL, Zhang HL, Zheng XP, Zhou QJ, Yang Y, Chen XQ, Du AF (2016). Structural and functional characterization of a novel gene, *Hc-daf-22*, from the strongylid nematode *Haemonchus contortus*.. Parasit Vectors.

[38] Pfaffl MW (2001). A new mathematical model for relative quantification in real-time RT-PCR.. Nucleic Acids Res.

[39] Kotze AC, Bagnall NH (2006). RNA interference in *Haemonchus contortus*: suppression of beta-tubulin gene expression in L3, L4 and adult worms *in vitro*.. Mol Biochem Parasitol.

[40] Wang Y, Fang R, Yuan Y, Pan M, Hu M, Zhou Y, Shen B, Zhao J (2016). Identification of host proteins, Spata3 and Dkk2, interacting with *Toxoplasma gondii* micronemal protein MIC3.. Parasitol Res.

