# Supplementary material for: A novel BR-SMAD is required for larval development in barber's pole worm Haemonchus contortus
Source: Microb Cell. 2020 Dec 23;8(2):57–64. doi: 10.15698/mic2021.02.742 (PMC7841850; doi:10.15698/mic2021.02.742)
Supplement: Supplementary file 1 [file mic-08-057-s01.pdf]

Table S1. Oligonucleotides used in this study

**Primers**	**Sequences (5'-3')**
For amplification of CDS	
*Hc*SMA2-F	ATGAGCATACAAACGAAG
*Hc*SMA2-R	ATGAGCATACAAACGAAG
For the preparation of dsRNA	
*Hc*sma2i-F1	**TAATACGACTCACTATAGGGAGA**CATTGGGCGACAATCAGCTA
*Hc*sma2i-R1	AAGCTTATGGATGAGATCGGTCGAGG
*Hc*sma2i-F2	AAGCTTCATTGGGCGACAATCAGCTA
*Hc*sma2i-R2	**TAATACGACTCACTATAGGGAGA**ATGGATGAGATCGGTCGAGG
*Bt*cry1Ac-F1	**TAATACGACTCACTATAGGG**CCAATACAGTACCAGCTACAG
Btcry1Ac-R1	GGATCCGATTCGGCTCTCCACAC
*Bt*cry1Ac-F2	GGATCCCCAATACAGTACCAGCTACAG
*Bt*cry1Ac-R2	**AATACGACTCACTATAGGG**GATTCGGCTCTCCACAC
For real-time PCR	
rt*Hc*sma2-F	ATCCCACCAGGAATTCGACG
rt*Hc*sma2-R	CATCGTCCACGTGCATTTGA
Tubulin-F	TGTTCCATCACCCAAGGTATCC
Tubulin-R	TGACAGACACAAGGTGGTTGAGAT
18S-F	AATGGTTAAGAGGGACAATTCG
18S-R	CTTGGCAAATGCTTTCGC

For bimolecular fluorescence complementation plasmids

*Hc*daf8-HA-F	TGAACCGTCAGATCCGCTAGCCACCATGCGATCCTTGTTCGAATC
*Hc*daf8-HA-R	GCACATCGTAAGGATATCTCGAGCCCGTAAATGAGGAATGTGCCA
*Hc*sma2-Myc-F	TGAACCGTCAGATCCGCTAGCCACCATGAGCATACAAACGAAGTT
*Hc*sma2-Myc-R	TGATCAGCTTCTGCTCGCCGATCGCTGAAATGGATGAGATCGGTC

Underlined base pairs represent restriction sites, and those in bold letters indicate the T7 promoter site.

Table S2. Sequences of siRNA used for RNA interference (5'-3')

siRNAs	Sense strand	Antisense strand	Position of the target sequence
siRNA-136	GCCAGUCUGGAAUUCGCAUTT	AUGCGAAUUCCAGACUGGCTT	136-154
siRNA-625	CCAGAACAUUGGGCGACAATT	UUGUCGCCCAAUGUUCUGGTT	625-644
siRNA-1130	GCUGGAUCGAAAUUCAUUUTT	AAAUGAAUUUCGAUCCAGCTT	1130-1149
Negative siRNA	UUCUCCGAACGUGUCACGUTT	ACGUGACACGUUCGGAGAATT	/
